# Genomic characterization of nine *Clostridioides difficile* strains isolated from Korean patients with *Clostridioides difficile* infection

**DOI:** 10.1186/s13099-021-00451-3

**Published:** 2021-09-16

**Authors:** Seung Woo Ahn, Se Hee Lee, Uh Jin Kim, Hee-Chang Jang, Hak-Jong Choi, Hyon E. Choy, Seung Ji Kang, Seong Woon Roh

**Affiliations:** 1 Microbiology and Functionality Research Group, World Institute of Kimchi, 86, Kimchi-ro, Nam-gu, 61755 Gwangju, Republic of Korea; 2 Department of Infectious Diseases, Chonnam National University Hospital, 61469 Gwangju, Republic of Korea; 3 Department of Microbiology, Chonnam National University Medical School, 61469 Gwangju, Republic of Korea

**Keywords:** *Clostridioides difficile*, *Clostridioides difficile* infection, Comparative genomics, Pathogenic features, Toxin A and toxin B, Antibiotic resistance gene

## Abstract

**Background:**

*Clostridioides difficile* infection (CDI) is an infectious nosocomial disease caused by *Clostridioides difficile*, an opportunistic pathogen that occurs in the intestine after extensive antibiotic regimens.

**Results:**

Nine *C. difficile* strains (CBA7201–CBA7209) were isolated from nine patients diagnosed with CDI at the national university hospital in Korea, and the whole genomes of these strains were sequenced to identify their genomic characteristics. Comparative genomic analysis was performed using 51 reference strains and the nine isolated herein. Phylogenetic analysis based on 16S rRNA gene sequences confirmed that all 60 *C. difficile* strains belong to the genus *Clostridioides*, while core-genome tree indicated that they were divided into five groups, which was consistent with the results of MLST clade analysis. All strains were confirmed to have a clindamycin antibiotic resistance gene, but the other antibiotic resistance genes differ depending on the MLST clade. Interestingly, the six strains belonging to the sequence type 17 among the nine *C. difficile* strains isolated here exhibited unique genomic characteristics for PaLoc and CdtLoc, the two toxin gene loci identified in this study, and harbored similar antibiotic resistance genes.

**Conclusion:**

In this study, we identified the specific genomic characteristics of Korean *C. difficile* strains, which could serve as basic information for CDI prevention and treatment in Korea.

**Supplementary Information:**

The online version contains supplementary material available at 10.1186/s13099-021-00451-3.

## Background

*Clostridioides difficile* is a major nosocomial pathogenic bacterium that poses a threat to public health worldwide [1]. An estimated 453,000 infections occur annually due to this organism; 15,000 deaths are directly attributable to infections caused by *C. difficile* in the United States [2]. In Europe and the UK, about 124,000 and 18,005 people are infected annually, respectively [3]. Recently, studies have revealed a high prevalence of CDI in East Asia, similar to that seen in Europe and North America [4]. In the Republic of Korea, the number of CDI patients per 100,000 people in a population increased from 1.43 in 2008 to 5.06 in 2011, while CDI-associated mortality increased from 0.14 to 0.35 [5]. These numbers are increasing yearly, especially in patients over 65 years old [6]. CDI is an opportunistic infectious disease caused by *C. difficile*, which grows and secretes toxins in the intestinal tract of the patient, resulting in a variety of symptoms including diarrhea and pseudomembranous colitis; it can be life-threatening [7]. CDI is treated with antibiotics such as cephalosporin, clindamycin, quinolone, metronidazole, and vancomycin [8, 9]. However, in some cases, diarrhea may recur or death may occur because these antibiotics can trigger infection [10].

*C. difficile* is a gram-positive, spore-forming, anaerobic, intestinal bacterium [2]. Recently, 16S rRNA gene sequence analysis of the *Clostridium* genus indicated that the similarity between *C. difficile* and *Clostridium hiranonis* is less than 97%; this has led to the reclassification of *Clostridium difficile* as *Clostridioides difficile* [11]. Secretion of toxin A (enterotoxin) and toxin B (cytotoxin) by *C. difficile* is mainly responsible for the intestinal inflammation resulting from CDI [12]. These two toxins inactivate host cell GTP-binding proteins and destroy the cytoskeleton, inducing apoptosis, severe inflammation, and intestinal cell damage [13–15]. Moreover, some hypervirulent strains (e.g. NAP1/ribotype 027) synthesize the actin-ADP-ribosylating toxin known as binary toxin *C. difficile* transferase (CDT), which leads to the depolymerization of actin filaments, disrupting the actin cytoskeleton in the cytosol [16–19]. Therefore, the toxins A, B, and CDT can cause severe CDI symptoms [20].

The first complete genome sequence of *C. difficile* reported was strain 630 [21]. Subsequently, the genomic information of a variety of *C. difficile* strains has been reported and deposited (https://www.ncbi.nlm.nih.gov/genome/genomes/535). In addition to uncovering the genetic and evolutionary diversity of *C. difficile* strains [22, 23], virulence factors of these strains, such as toxins, antibiotic resistance, mobility, and adhesion have been also investigated through a comparative genomic analysis [24]. Although there have been many studies on *C. difficile* strains isolated from various patients with CDI worldwide, there have been few genomic studies conducted on *C. difficile* strains in the Republic of Korea. Thus, this study aimed to investigate the unique genomic characteristics of nine *C. difficile* strains isolated from South Korean patients through a comparative genomic analysis with previously characterized strains.

## Methods

### Ethical statement and sample collection

Stool samples were collected from nine patients diagnosed with CDI who visited the Department of infectious disease, Chonnam national university hospital in Gwangju or Hwasun, Republic of Korea. The study protocol was approved by the institutional review boards of the Republic of Korea centers for disease control and Prevention [IRB file no. CNUH-2017-161 and CNUHH-2017-076]. Written informed consent was obtained from all participants. The characteristics of all patients who agreed to fecal sampling after confirmation of CDI and the list of strains isolated from each fecal sample, as well as the prescribed antibiotics for each patient before fecal sampling, are summarized in Table 1.

**Table 1:** Patients with CDI who provided fecal samples for *C. difficile* isolation and the prescribed antibiotics

Subject no	Subject name	Age (y)	Sex	*C. difficile* strain isolated	Prescribed antibiotics class (product name)
1	Hwasun03	85	M	CBA7201	Nitroimidazole (Furacinil, Trizel, Furacinil), cephalosporin (Zenocef)
2	Gwangju02	79	M	CBA7202	Nitroimidazole (Furacinil), cephalosporin (Cetrazol), glycopeptide (Targocid, Vancozin, IV Vancomycin), fluoroquinolone (Levofexin), linezolid (Zyvox), penicillin (Tazoperan), polymyxin (Colis), glycylcycline (Tygacil), sulfonamide (Ceptrin), azoles (Diflucan), polyene antimycotic (PMS-Nystatin)
3	Hwasun11	63	F	CBA7203	Nitroimidazole (Furacinil), cephalosporin (Cetrazole)
4	Gwangju06	79	F	CBA7204	Nitroimidazole (Furacinil), glycopeptide (Vancocin, Teiconin, Vancomycin), penicillin (Tazoperan)
5	Hwasun12	61	F	CBA7205	Nitroimidazole (Furacinil), cephalosporin (Cetrazol), glycopeptide (Teiconin, IV Vancomycin), fluoroquinolone (Cravit), penicillin (Tazoperan), sulfonamide (Sevatrim, Septrin), carbapenem (Meropen), macrolide (Zithromax)
6	Hwasun13	77	F	CBA7206	Nitroimidazole (Furacinil), fluoroquinolone (Cravit), penicillin (Tazoperan), macrolide (Klaricid)
7	Hwasun15	65	M	CBA7207	Nitroimidazole (Furacinil), cephalosporin (Pacetin), glycopeptide (IV Vancomycin)
8	Hwasun16	62	F	CBA7208	Nitroimidazole (Trizel), glycopeptide (Vancomycin), penicillin (Augmentin)
9	Hwasun18	79	F	CBA7209	Cephalosporin (Cefazolin, Maxipime), glycopeptide (Teiconin, IV Vancomycin)

### Culture conditions and identification of *C. difficile* isolated from CDI participants

Collected stool samples were treated with chloroform for efficient isolation of *C. difficile* [25] as chloroform selectively isolates *C. difficile* by removing non-spore forming bacteria in the stool samples. Chloroform (60 µL; concentration, 3%) was added to filtered PBS (1740 µL), after which 200 µL of the stool sample was added. The mixed samples were suspended in a shaking incubator for 1 h at 37 °C, after which chloroform was evaporated with N_2_ gas (Automated gas distribution workstation; Raontech, Gwangju, Republic of Korea), followed by culturing in Cycloserine-Cefoxitin Fructose Agar (CCFA) medium, which is an enriched selective and differential medium for the isolation and presumptive identification of *C. difficile*. CCFA medium consists of 40.0 g proteose peptone, 5.0 g sodium phosphate dibasic, 1.0 g potassium phosphate monobasic, 2.0 g sodium chloride, 6.0 g fructose, 15.0 g agar, 9.0 mg neutral red, 500.0 mg cycloserine (10.0% solution), and 15.6 mg cefoxitin (1.56% solution). Cycloserine inhibits the growth of Gram-negative bacteria, while cefoxitin inhibits the growth of both Gram-positive and -negative organisms [26]. *C. difficile* can be resistant to cefoxitin, and CCFA with cefoxitin is an initial formulation that can be used to isolate *C. difficile* strains [26–28]. The samples were cultured under anaerobic conditions in an anaerobic chamber (BACTRON anaerobic chamber; Sheldon Manufacturing, Inc., Cornelius, OR) containing an atmosphere of 90% N_2_, 5% H_2_, and 5% CO_2_, at 37 °C. After incubation for more than 48 h, single colonies were obtained and transferred at least three times until considered pure. The 16S rRNA gene of the pure cultures was amplified using colony PCR with the bacterial universal primers 27F (5ʹ-GTTTGATCCTGGCTCAG-3ʹ) and 1492R (5ʹ-TACGGYTACCTTGTTACGACTT-3ʹ) [29]; identification of the cultures was performed based on the 16S rRNA gene sequences obtained from Sanger sequencing, with the EzBioCloud Database [30]. For comparative genomic analysis, nine *C. difficile* strains (designated CBA7201–CBA7209) were selected from each patient with CDI.

### Genomic DNA extraction and whole-genome sequencing analysis

For genome sequencing of the selected nine *C. difficile* strains, cells were cultivated to the stationary phase in brain heart infusion (BD Biosciences, Franklin Lakes, NJ) broth medium at 37 °C and harvested by centrifugation. Genomic DNA was extracted and purified using MagAttract HMW DNA Kit (Qiagen, Hilden, Germany) and MG Genomic DNA Purification Kit (MGmed, Seoul, Republic of Korea), followed by quantification with PicoGreen (Invitrogen, Carlsbad, CA). The genomes were then sequenced with the PacBio RS II System using single-molecule real-time (SMRT) sequencing technology based on a 20 kb library (Pacific Biosciences, Menlo Park, CA). Assembly was performed using the hierarchical genome assembly process 2 protocol in PacBio SMRT analysis v2.3.0 [31]. The whole-genome sequences of strains CBA7201–CBA7209 were deposited in GenBank (accession numbers QKRF00000000, QLNX00000000, QKRE00000000, CP029566, QLNY00000000, QLNZ00000000, QLOA00000000, QKRD00000000, and QLOB00000000, respectively) and automatically annotated by the NCBI prokaryotic genome annotation pipeline [32]. A total of 60 *C. difficile* strains including the nine strains isolated herein and 51 strains from the NCBI GenBank, were used for comparative genomic analysis. Data were obtained using the *Clostridium difficile* MLST Databases of PubMLST for sequence types (STs) and multi-locus sequence typing (MLST) clades (Table 2) [33].

**Table 2:** General features of the 60 *C. difficile* genomes employed in this study^a^

Strain name (accession no.)	Genome status^a^ (no. of contigs)	Total size^a^ (Mb)	No. of genes^a^	G + C content^a^(%)	MLST clade^b^	STs^b^	Sampling country
***C. difficile* CBA7201 (QKRF00000000)**^**c**^	**C (3)**	**4.34**	**4,107**	**28.8**	**1**	**17**	**Korea**
***C. difficile* CBA7202 (QLNX00000000)**^**c**^	**D (3)**	**4.40**	**4,204**	**28.8**	**1**	**17**	**Korea**
***C. difficile* CBA7203 (QKRE00000000)**^**c**^	**D (5)**	**4.39**	**4,189**	**28.8**	**1**	**17**	**Korea**
***C. difficile* CBA7204 (CP029566)**^**c**^	**C (1)**	**4.04**	**3,744**	**28.5**	**1**	**203**	**Korea**
***C. difficile* CBA7205 (QLNY00000000)**^**c**^	**D (7)**	**4.40**	**4,176**	**28.9**	**1**	**17**	**Korea**
***C. difficile* CBA7206 (QLNZ00000000)**^**c**^	**D (7)**	**4.16**	**3,911**	**28.7**	**1**	**8**	**Korea**
***C. difficile* CBA7207 (QLOA00000000)**^**c**^	**D (2)**	**4.33**	**4,101**	**28.8**	**1**	**17**	**Korea**
***C. difficile* CBA7208 (QKRD00000000)**^**c**^	**C (2)**	**4.08**	**3,786**	**28.5**	**1**	**4**	**Korea**
***C. difficile* CBA7209 (QLOB00000000)**^**c**^	**D (2)**	**4.40**	**4,191**	**28.8**	**1**	**17**	**Korea**
*C*. *difficile* 630 (AM180355-6) *C*. *difficile* DSM 27639 (CP011847) *C*. *difficile* DSM 29745 (CP019857) *C*. *difficile* DSM 29688 (CP019858) *C*. *difficile* W0022a (CP025046) *C*. *difficile* DSM 29632 (CP019860) *C*. *difficile* 08ACD0030 (CP010888) *C*. *difficile* BR81 (CP019870) *C*. *difficile* Mta-79 (CP042267) *C*. *difficile* DSM 28666 (CP012321) *C*. *difficile* DSM 29637 (CP016106) *C*. *difficile* W0023a (CP025045) *C*. *difficile* FDAARGOS_267 (CP020424-6) *C*. *difficile* DH/NAP11/106/ST-42 (CP022524) *C*. *difficile* W0003a (CP025047) *C*. *difficile* 020477 (CP028524) *C*. *difficile* 020709 (CP028529) *C*. *difficile* QCD-63q42 (CM000637) *C*. *difficile* DSM 1296^ T^ (CP011968-9) *C*. *difficile* DSM 27638 (CP011846) *C*. *difficile* DSM 27640 (CP011848) *C*. *difficile* CD-17-01474 (CP026591) *C*. *difficile* R0104a (CP025044) *C*. *difficile* 08-00495 (CP026594) *C*. *difficile* 10-00253 (CP026598) *C*. *difficile* 12-00011 (CP026595) *C*. *difficile* 09-00072 (CP026599) *C*. *difficile* 10-00,078 (CP026597) *C*. *difficile* 10-00071 (CP026596) *C*. *difficile* 12-00008 (CP026593) *C*. *difficile* CD-10-00484 (CP026592) *C*. *difficile* CD196 (FN538970) *C*. *difficile* QCD-66c26 (CM000441) *C*. *difficile* DSM 102860 (CP020379) *C*. *difficile* DSM 102859 (CP020378) *C*. *difficile* VL_0104 (FAAJ00000000) *C*. *difficile* VL_0391 (FALK00000000) *C*. *difficile* ZJCDC-S82 (JYNK00000000) *C*. *difficile* CDT4 (CP029152-3) *C*. *difficile* DSM 29627 (CP016102) *C*. *difficile* DSM 28670 (CP012312) *C*. *difficile* CD161 (CP029154-6) *C*. *difficile* DSM 28669 (CP012323) *C*. *difficile* DSM 29629 (CP016104) *C*. *difficile* M68 (FN668375) *C*. *difficile* DSM 29747 (CP019864) *C*. *difficile* 12038 (CP033214-5) *C*. *difficile* CD10010 (CP033213) *C*. *difficile* M120 (FN665653) *C*. *difficile* CD21062 (CP033216-7) *C*. *difficile* DSM 29,020 (CP012325)	C (2) C (1) C (1) C (1) C (1) C (1) C (1) C (1) C (1) C (1) C (1) C (1) C (3) C (1) C (1) C (1) C (1) D (28) C (2) C (1) C (1) C (1) C (1) C (1) C (1) C (1) C (1) C (1) C (1) C (1) C (1) C (1) D (15) C (1) C (1) D (261) D (1,092) D (20) C (2) C (1) C (1) C (3) C (1) C (1) C (1) C (1) C (2) C (1) C (1) C (2) C (1)	4.29 4.26 4.24 4.22 4.18 4.17 4.16 4.12 4.12 4.12 4.11 4.11 4.28 4.08 4.07 4.14 4.09 4.44 4.28 4.22 4.22 4.20 4.19 4.17 4.12 4.11 4.11 4.11 4.11 4.11 4.11 4.11 4.12 4.25 4.24 4.06 4.16 4.22 4.28 4.20 4.19 4.47 4.13 4.11 4.3 4.07 4.07 4.04 4.04 4.10 4.13	3981 4026 3975 4041 3942 3891 3907 3813 3844 3829 3844 3804 4049 3743 3765 3873 3792 4213 3984 3966 3964 3939 3924 3902 3,844 3,845 3,842 3,845 3843 3842 3843 3807 3774 4071 4050 3,859 3939 3934 4035 3916 3962 4277 3908 3802 4025 3809 3822 3785 3756 3898 3905	29.0 29.1 29.0 28.9 28.9 28.6 28.8 28.7 28.7 29.0 28.6 28.7 28.7 28.6 28.6 28.8 28.5 28.6 28.7 29.0 29.0 28.9 28.7 28.7 28.6 28.6 28.6 28.6 28.6 28.6 28.6 28.6 28.5 29.0 29.0 28.7 29.0 29.1 28.8 28.8 28.8 28.8 28.8 28.6 28.9 29.1 28.8 28.7 28.7 28.9 29.2	1 1 1 1 1 1 1 1 1 1 1 1 1 1 1 1 1 1 1 2 2 2 2 2 2 2 2 2 2 2 2 2 2 3 3 3 3 3 4 4 4 4 4 4 4 5 5 5 5 5 5	54 54 3 15 2 103 2 42 34 48 83 42 3 42 8 110 21 3 3 1 1 1 1 1 1 1 1 1 1 1 1 1 1 5 5 201 201 5 37 37 38 37 109 39 37 11 11 11 11 11 11	Switzerland Germany Germany Germany USA Indonesia Canada Korea USA Ghana Indonesia USA USA USA USA USA USA Canada UK Germany Germany Germany USA Germany Germany Germany Germany Germany Germany Germany Germany France Canada Germany Germany Canada Canada China China Indonesia Ghana China Ghana Indonesia Ireland Germany China China UK China Indonesia

^a^Bioinformatic genome analysis was carried out using the NCBI prokaryotic genome annotation pipeline (http://www.ncbi.nlm.nih.gov/genome/annotation_prok/)

^b^MLST clade and ST information was obtained using PubMLST analysis (https://pubmlst.org/cdifficile/)

^c^Genomes sequenced in this study are highlighted in bold

^d^Genome status: *D* draft genome sequence, *C* complete genome sequence

### Phylogenetic analyses of *C. difficile* genomes based on 16S rRNA gene and whole genome sequences

A phylogenetic tree based on the 16S rRNA gene sequences was constructed to infer the phylogenetic relationships among the 60 strains. The 16S rRNA gene sequences were aligned using the fast secondary-structure aware infernal aligner in the ribosomal database project [34]. For pan-genome and core-genome analysis between the 60 strains, the bacterial pan-genome analysis pipeline ver. 1.3 was used [35]. The core-genome of all *C. difficile* strains was extracted through all-against-all comparisons using the USEARCH (ver. 9.0) with a 50% sequence identity cut-off and their concatenated nucleotide sequences were aligned using the MAFFT program (ver. 7.407) available in the Roary pipeline [36]. The phylogenetic trees based on the aligned 16S rRNA gene sequences and the concatenated common gene sequences were constructed using the neighbor-joining (NJ) algorithm in the MEGA7 software [37]. Average nucleotide identity (ANI) and in silico DNA-DNA hybridization (DDH) analysis were used to assess the relatedness among the 60 *C. difficile* strains and two reference strains (*Clostridioides mangenotii* DSM 1289^T^ and *Clostridium hiranonis* TO-931). The pair-wise ANI value among the genomes was calculated using a stand-alone OrthoANI software [38]. Pair-wise in silico DDH was calculated using the genome-to-genome distance calculator 2.1 [39]. In silico DDH values among the *C. difficile* strains were calculated and visualized using the GENE-E software (https://software.broadinstitute.org/GENE-E/).

### Functional and pathogen-associated gene analysis of *C. difficile* strains

The amino acid sequences of 60 *C. difficile* strains were analyzed using GhostKOALA based on the Kyoto Encyclopedia of Genes and Genomes (KEGG) database to obtain predicted protein annotation information [40]. The resulting KEGG Orthology (KO) numbers were summarized and visualized on the KEGG pathway using iPath2.0 [41]. Flagella assembly, pathogenicity locus (PaLoc) (*tcdRBEAC*), and binary toxin (*cdtAB*) genes in *C*. *difficile* strains were confirmed through BLASTP analyses using the reference protein sequences available in closely related *C. difficile* strains. Antibiotic resistance genes were identified using the Comprehensive Antibiotic Resistance Database (CARD) [42]. Nucleotide sequence similarity was calculated using EMBOSS Water, the pairwise sequence alignment tool provided by EMBL-EBI (https://www.ebi.ac.uk/Tools/psa/), against the nucleotide sequence of *C. difficile* 630 strain [43].

### Quality assurance

Before genomic DNA extraction, the single colonies of each of strain CBA7201–CBA7209 were transferred three times in CCFA medium to obtain pure single colony. After obtaining the whole genome sequence of strain CBA7201–CBA7209, the sequences of the 16S rRNA genes, extracted using RNAmmer 1.21 server, were confirmed using the EzBioCloud database.

## Results and discussion

### Isolation and phylogenetic relatedness of *C. difficile* strains

A total of nine *C. difficile* strains (CBA7201–CBA7209) were isolated and selected for genomic analysis, as considering different isolation source and 16S rRNA gene sequence homology (Table1). After incubation for 24 h under anaerobic conditions on CCFA medium at 37 °C, the colony morphology of *C. difficile* strains appeared white or grayish-white and had an irregular radial shape. Although *C. difficile* is an anaerobic bacterium, strains CBA7201–CBA7209 were viable when exposed to aerobic conditions for 24 h. *C. difficile* can resist environmental stressors, such as exposure to oxygen, through spore formation. This stress-resistant feature may aid the spread of *C. difficile* in various environments [44, 45].

**Fig. 1:**
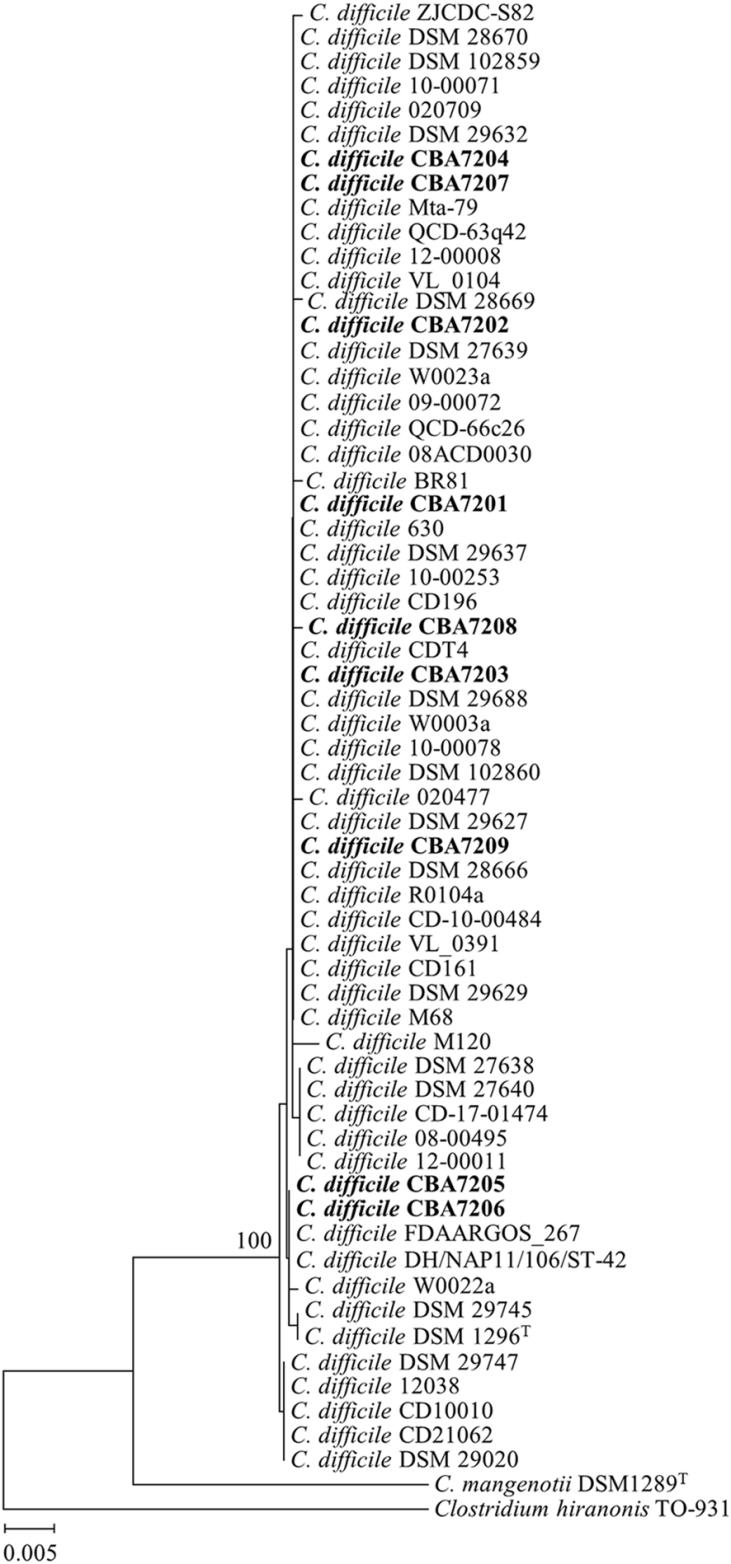
Phylogenetic tree based on 16S rRNA gene sequences. 16S rRNA gene-based phylogenetic tree constructed using NJ algorithm showing phylogenetic relationships between 60 *C. difficile* strains with *C. mangenotii* DSM 1289^T^ and *Clostridium hiranonis* TO-931. Strain CBA7201–CBA7209 isolated from Korean patients with CDI are highlighted in bold

To assess the phylogenetic relationship between the *C. difficile* strains, a phylogenetic tree based on 16S rRNA gene sequences was constructed using the nine *C. difficile* isolates (CBA7201–CBA7209), 51 strains from GenBank, and two other closely related species, *C. mangenotii* DSM 1289^T^ and *Clostridium hiranonis* TO-931 (Fig. 1). For strains CBA7201–CBA7209, none were sufficiently different to be classified as strains from other species, as all 60 strains of *C. difficile* tested were grouped into one lineage that was distinct from the two outgroup strains [46, 47]. The 60 strains showed 99.9% 16S rRNA gene sequence similarity with the type strain *C. difficile* DSM 1296^T^, and were thus, classified as *C. difficile.* However, the *C. difficile* strains and *Clostridium hiranonis* TO-931 were distinct, supporting the reclassification of *C. difficile* under the genus *Clostridioides* [11]. *C. difficile* was first classified as *Clostridium* because its characteristics (anaerobic, Gram-positive, and spore-forming) were similar to those of other *Clostridium* species. However, further studies using molecular methods indicated a diversity of organisms in the genus *Clostridium*, and 16S rRNA phylogenetic analysis confirmed that *C. difficile* had less than 97% similarity with other species from the genus *Clostridium*. Currently, the genus *Clostridioides* includes two species, *C. difficile* and *C. mangenotii* [11, 48].

Phylogenetic analysis based on the 16S rRNA gene, a molecular ecological marker, showed no differences among the 60 *C. difficile* strains, suggesting a limitation to the information this marker gene can provide [49]. To overcome this, comparative genome analysis with their entire genomes is conducted to identify the characteristics of various *C. difficile* strains [50, 51].

### General features of the *C. difficile* genomes

The complete genomes of the nine *C. difficile* strains isolated in this study were obtained by performing whole-genome sequencing using the PacBio RS II System. The *C. difficile* CBA7201–CBA7209 genomes and 51 additional *C. difficile* genomes available in GenBank were compared and their general characteristics described in Table 2. The average genome size and gene numbers were 4.18 ± 0.1 Mb and 3927 ± 131, respectively. The genome of *C. difficile* CBA7204 was the smallest (4.04 Mb), whereas that of *C. difficile* CD161, which was isolated in China and is known as a hypervirulent strain, was the largest (4.47 Mb) [52]. The G + C content of the *C. difficile* genomes ranged from 28.5% to 29.2%. The MLST scheme for *C. difficile* is based on the following seven highly conserved housekeeping genes: adenylate kinase (*adk*), ATP synthase subunit alpha (*atpA*), 1-deoxy-D-xylulose 5-phosphate reductoisomerase (*dxr*), serine hydroxymethyltransferase (*glyA*), recombinase A (*recA*), superoxide dismutase (*sodA*), and triosephosphate isomerase (*tpi)*. STs are determined according to a combination of these seven housekeeping genes and are classified into five MLST clades (clade 1–5) [53]. To investigate genomic diversity, MLST clades and STs of the 60 strains of *C. difficile* were assigned using PubMLST and are listed in Table 2. A number of *C. difficile* strains, including CBA7201–CBA7209 and 19 reference strains, were assigned to MLST clade 1, which is consistent with the most frequently identified *C. difficile* strains worldwide [54]. Here, the MLST clade 1 belonging to the 28 strains, included 15 kinds of STs (ST2, ST3, ST4, ST8, ST15, ST17, ST21, ST24, ST42, ST48, ST54, ST83, ST103, ST110, and ST203) and had the most types of STs, making it the most diverse in terms of PCR ribotype (RT), which consists of a combination of STs and toxic genes encoding toxins A, B, and CDT. Among them, ST17 (*C. difficile* CBA7201, CBA7202, CBA7203, CBA7205, CBA7207, CBA7209) is associated with RT018 [55] which is the most prominent ribotype reported in hospitals in the Republic of Korea [56] and Japan [57–59]. RT018 is highly contagious and has been found to account for more than 95% CDI relapse cases. A study also revealed that patients with the RT018 were older than those with other RTs, and there was an association between the infectious RT018 and age [60]. Here, the most identified ST was ST1, which was identified in 14 out of the 60 strains and belongs to MLST clade 2; ST1 has been reported to be associated with an increased mortality rate of communicable diseases in North America and Europe [61–63].

### Phylogenetic relatedness of *C. difficile* strains based on pan- and core-genome analysis

**Fig. 2:**
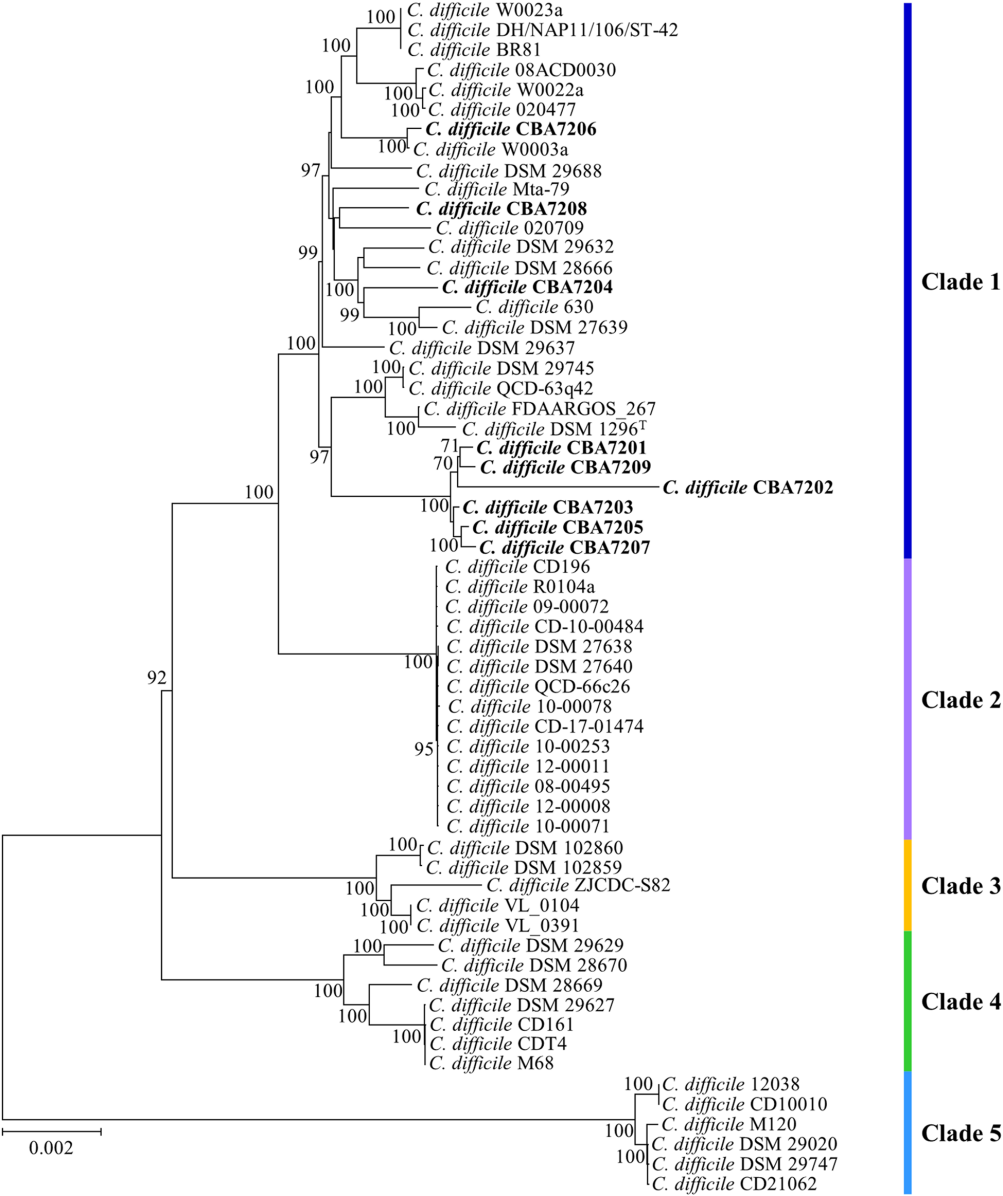
Phylogenetic tree based on core-gene sequences. Phylogenetic tree generated from the amino acid sequences of the 60 *C. difficile* core genomes showing the relationship between each strain. The MLST clade and ST data were obtained from the PubMLST analysis. The vertical bar on the right indicates the MLST clade to which each *C. difficile* strain belongs. Strain CBA7201–CBA7209 isolated from Korean patients with CDI are highlighted in bold

Pan-genome analysis is a useful tool for effectively analyzing and expressing the genomic characteristics of bacteria. Through this analysis, we found 5,814 genes in pan-genome and 1,660 genes in core-genome across the 60 strains (Additional file 1: Figure S1), with the number of total unique genes being 643. The curve analysis based on the Heaps’ law regression model showed that the pan-genome was open (*B*_pan_ = 0.14), indicating that more sequenced strains are needed to capture the complete gene complement [64]. The number of accessory and unique genes in the 60 strains are listed in Additional file 2: Table S1. OrthoANI values showed the pairwise relatedness of the nine *C. difficile* strains isolated in this study with the reference strains (Additional file 2: Table S2). This result suggests that the nine Korean strains are not part of a common clone. Moreover, the unique genes and different OrthoANI values reflect the existence of evolutionary differences and ecological niches for each strain that help it adapt to varying environmental stressors. To further investigate the phylogenic relationships among the *C. difficile* strains, we constructed a phylogenetic tree based on the amino acid sequences of 1,660 core genes. Despite being the same species, the core gene-based phylogenetic tree indicated that the strains were divided into five groups (Fig. 2). Unlike the 16S rRNA-based phylogenetic tree, the difference between the strains in the core gene-based phylogenetic tree was more clearly distinguishable. This classification is consistent with the hierarchical classification based on the in silico DDH (Additional file 1: Fig. S2), as well as clustering based on the MLST clade (Fig. 2; vertical bar on the right side). These results indicate that MLST clade-based classification using the seven conserved housekeeping genes is a very efficient method for distinguishing *C. difficile* strains. According to the core gene-based phylogenetic tree analysis, a 28-genomes group containing *C. difficile* strains CBA7201–CBA7209, and strain 630 was consistent with MLST clade 1. Among them, *C. difficile* CBA7201, CBA7202, CBA7203, CBA7205, CBA7207, and CBA7209 formed a distinct group, indicating similar genomic features. The other groups were clustered into MLST clades 2, 3, 4, and 5. Strains belonging to clade 5 in the core gene phylogenetic tree were significantly divergent from the other clade 1–4 strains, indicating that clade 5 strains, which were all ST11 (Table 2), may have undergone different evolutionary processes [50, 65].

### Functional category analysis of *C. difficile* genome

**Fig. 3:**
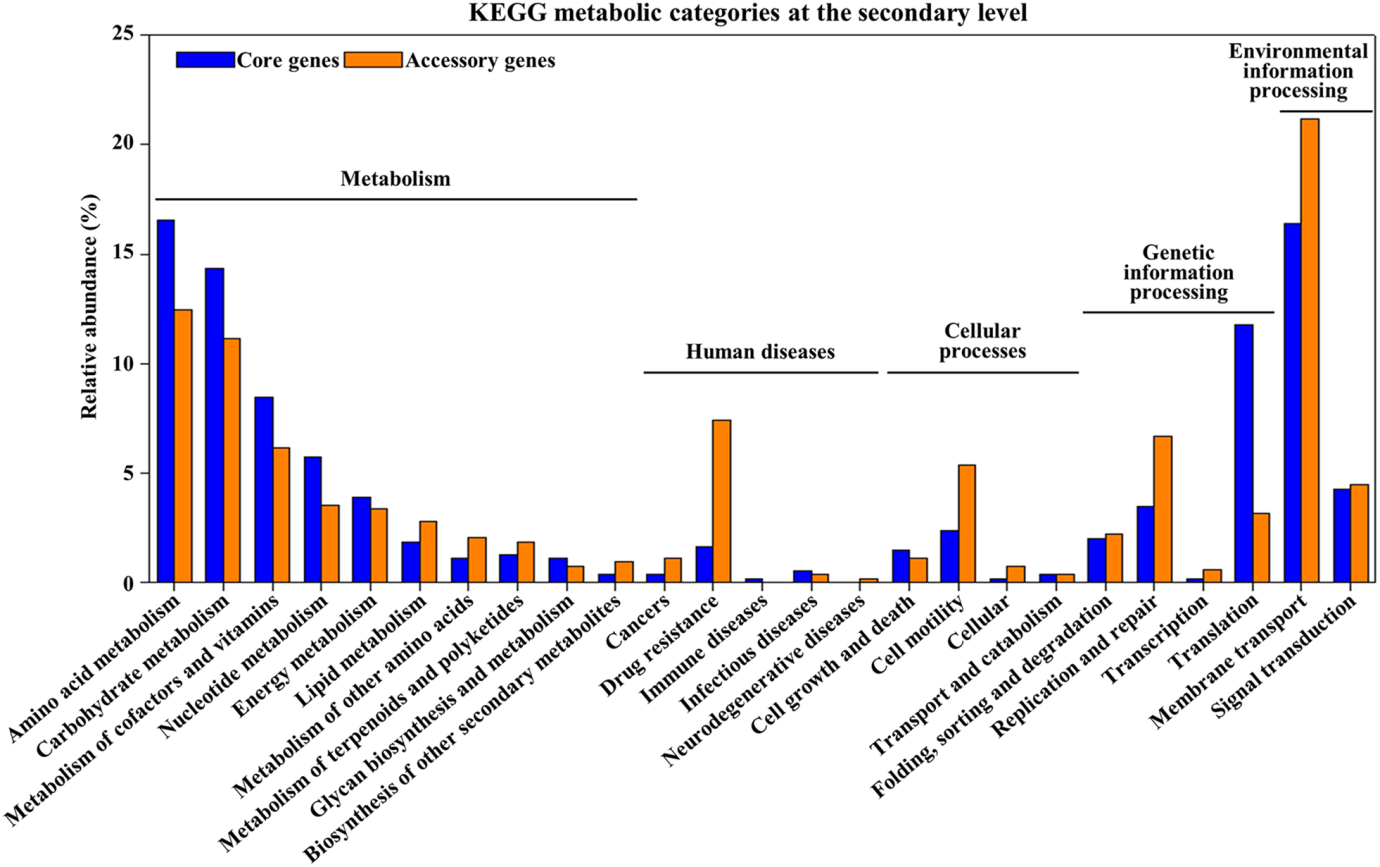
KEGG functional categories at the secondary levels. Comparison of KEGG functional categories at the secondary levels in core and accessory genes of the 60 *C. difficile* genomes

To identify the general metabolic diversity, functional features, and virulence factors of the *C. difficile* strains, KEGG analysis was carried out using the core and accessory genes (Fig. 3). All core and accessory genes were most frequently classified under amino acid metabolism, carbohydrate metabolism, and membrane transport. The terms amino acid metabolism and carbohydrate metabolism were more abundant in the core genes than in the accessory genes, which is a common feature of *C. difficile*. In contrast, membrane transport (phosphor transferase system, ABC transporter, and bacterial secretion system) was relatively abundant in the accessory genes, indicating that the bacteria can absorb or secrete various substances depending on the strain. Among the genes assigned to the human disease category, drug resistance genes were relatively abundant in the accessory genes, suggesting that *C. difficile* strains have been exposed to various antibiotics or antibiotic-resistant genes and that their antibiotic resistance was obtained differently depending on the strains [66].

Using CARD, antibiotic resistance-related genes of the 60 *C. difficile* strains are summarized in Table 3 and Additional file 2: Table S3. The 60 *C. difficile* strains contained at least one resistance gene among the 20 Antibiotic Resistance Ontology (ARO) terms; this gene is associated with resistance to erythromycin and clindamycin, which belong to the macrolide and lincosamide class of antibiotics, respectively (ARO term: *C. difficile* 23S rRNA with mutation conferring resistance to erythromycin and clindamycin). Most cases of resistance to these antibiotics can be associated with alterations in nucleotides of 23S rRNA, a component of the large ribosomal subunit [67]. The resistance of *C. difficile* strains to erythromycin and clindamycin has been confirmed in previous CDI-related studies [27]. Moreover, clindamycin has been reported to pose a risk as it promotes CDI; thus, care must be taken when prescribing it [66].

**Table 3:** Antibiotics resistance-related genes of *C. difficile* strains based on the CARD

ARO term	AMR gene family	Drug class	Resistance mechanism	*C. difficile* strain
*C. difficile* 23S rRNA with mutation conferring resistance to erythromycin and clindamycin	23S rRNA with mutation conferring resistance to macrolide antibiotics	Macrolide, lincosamide	Antibiotic target alteration	All 60 strains
*ErmB*	Erm 23S ribosomal RNA methyltransferase	Macrolide, lincosamide, streptogramin	Antibiotic target alteration	**CBA7201**–**CBA7203**, **CBA7205**, **CBA7207**, **CBA7209**, 630, DSM 27639, DSM 29745, DSM 29688, DSM 28666, W0023a, R0104a, CD161, DSM 28669, M68
*vanRG*	Glycopeptide resistance gene cluster, *vanR*	Glycopeptide	Antibiotic target alteration	**CBA7201**–**CBA7209**, 630, DSM 27639, DSM 29745, DSM 2,688, W0022a, DSM 29632, 08ACD0030, BR81, Mta-79, DSM 28666, DSM 29637, W0023a, FDAARGOS267, DH/NAP11/106/ST-42, W0003a, 20477, 20709, QCD-63q42, DSM 1296^T^, DSM 27638, DSM 27640, CD-17-01474, R0104a, 08-00495, 10–00253, 12-00011, 09-00072, 10-00078, 10-00071, 12-00008, CD-10-–00484, CD196, QCD-66c26
*vanXYG*	Glycopeptide resistance gene cluster, *vanXY*	Glycopeptide	Antibiotic target alteration	**CBA7201**, **CBA7203**–**CBA7209**, 630, DSM 27639, DSM 29745, DSM 29688, W0022a, DSM 29632, 08ACD0030, BR81, Mta-79, DSM 28666, DSM 29637, W0023a, FDAARGOS267, DH/NAP11/106/ST-42, W0003a, 20477, 20709, QCD-63q42, DSM 1296^T^, DSM 27,638, DSM 27,640, CD-17–01,474, R0104a, 08-00495, 10-00253, 12-00011, 09-00072, 10-00078, 10-00071, 12-00008, CD-10-00484, CD196, QCD-66c26
*Clostridioides difficile gyrA* conferring resistance to fluoroquinolones	Fluoroquinolone resistant *gyrA*	Fluoroquinolone	Antibiotic target alteration	**CBA7201–CBA7203**, **CBA7205–CBA7207**, **CBA7209**, DSM 29745, QCD-63q42, DSM 27638, DSM 27,640, CD-17-01474, R0104a, 08-00495, 10-00253, 12-00011, 10-00078, 10–00,071, 12-00008, CD-10-00484, QCD-66c26, VL 0104, VL 0391, CDT4, CD161, DSM 29747
*cdeA*	Multidrug and toxic compound extrusion (MATE) transporter	Fluoroquinolone, acridine dye	Antibiotic efflux	**CBA7209**, 630, DSM 29745
APH (2'')-If	APH(2'')	Aminoglycoside	Antibiotic inactivation	**CBA7201**–**CBA7203**, **CBA7205**, **CBA7207**, **CBA7209**, DSM 27638, DSM 27640
AAC(6')-Ie-APH(2'')-Ia	APH(2''), AAC(6')	Aminoglycoside	Antibiotic inactivation	630, ZJCDC-S82, CD161, M68
aad(6)	ANT(6)	Aminoglycoside	Antibiotic inactivation	DSM 29020
ANT(6)-Ia	ANT(6)	Aminoglycoside	Antibiotic inactivation	M120
ANT(6)-Ib	ANT(6)	Aminoglycoside	Antibiotic inactivation	M120
APH(3')-IIIa	APH(3')	Aminoglycoside	Antibiotic inactivation	CD21062
*catI*	Chloramphenicol acetyltransferase (CAT)	Phenicol	Antibiotic inactivation	CD21062
SAT-4	Streptothricin acetyltransferase (SAT)	Nucleoside	Antibiotic inactivation	DSM 29020
*tet*(40)	Major facilitator superfamily (MFS) antibiotic efflux pump	Tetracycline	Antibiotic efflux	DSM 29747, DSM 29020
*tet*(44)	Tetracycline-resistant ribosomal protection protein	Tetracycline	Antibiotic target protection	M120
*tet*(W/N/W)	Tetracycline-resistant ribosomal protection protein	Tetracycline	Antibiotic target protection	630
*tetB*(P)	Tetracycline-resistant ribosomal protection protein	Tetracycline	Antibiotic target protection	DSM 28669
*tetM*	Tetracycline-resistant ribosomal protection protein	Tetracycline	Antibiotic target protection	DSM 28666, CDT4, DSM 29627, CD161, M68, DSM 29747, M120, CD21062, DSM 29020
*tetO*	Tetracycline-resistant ribosomal protection protein	Tetracycline	Antibiotic target protection	CD21062

*ARO* antibiotic resistance ontology, *AMR* antimicrobial resistance

Strains CBA7201–CBA7209 isolated from Korean patients with CDI are highlighted in bold

Interestingly, *C. difficile* has different antibiotic resistance genes depending on the MLST clade. Most strains in MLST clade 1–3 possessed resistance genes against vancomycin (*vanR*_*G*_*, vanXY*_*G*_), a class of glycopeptide antibiotics. The *vanR*_*G*_ is a *vanR* variant and *vanXY*_*G*_ is a variant of *vanXY* found in the *vanG* gene cluster. Resistance of enterococci to glycopeptides was reported first [68], after which nine genotypes associated with this resistance were identified. The *vanG* is one of the nine genotypes (*vanA*, *vanB*, *vanC*, *vanD*, *vanE*, *vanG*, *vanL*, *vanM*, and *vanN*) that are involved in glycopeptide antibiotics resistance. It has been reported that similar *vanG* gene clusters also exist in *C. difficile*. The *vanG* phenotype is known to correspond to low-level resistance to vancomycin, which results from the acquisition of two *vanG* operons, *vanG1* and *vanG2*. The *vanR*_*G*_ gene is one of three regulatory genes of *vanG1*, while the *vanXY*_*G*_ gene is one of five effector genes of *vanG1* [69]. However, the similarity of the *vanR*_*G*_ and *vanXY*_*G*_ genes was 77.45%–77.87% and 58.82%–59.22% in CARD, respectively (Additional file 2: Table S3), suggesting that glycopeptide resistance by these two genes is not expected to function properly. Some of the strains belonging to the MLST clade 4 and 5 did not possess resistance genes of glycopeptide antibiotics, but had other resistance genes [ARO term: AAC(6')-Ie-APH(2'')-Ia, aad(6), ANT(6)-Ia, ANT(6)-Ib, APH(3')-IIIa, catI, SAT-4, tet(40), tet(44), tet(W/N/W), tetB(P), tetM, tetO)] specific for antibiotics that inhibit protein synthesis by 30S ribosomal subunits, such as aminoglycoside and tetracycline antibiotics [70, 71]. These findings indicate that different antibiotics can be prescribed depending on the MLST clade of the strain causing the infection. Compared with other strains, the nine *C. difficile* strains isolated from Korean patients with CDI possessed a higher number of antibiotic-resistance genes. In some cases, up to 11 antibiotics were prescribed for a patient, Gwangju02 (Table 1), and *C. difficile* strains with the genes resistant to the prescribed antibiotics were isolated from a total of seven patients. Therefore, more care should be taken when prescribing antibiotics to prevent persistent CDI and emergence of multidrug-resistant pathogens.

The cell motility category genes were relatively abundant among the accessory genes, indicating that genes associated with cell motility are different depending on the *C. difficile* strain. Most genes assigned to the flagellar assembly in the cell motility category were classified into the core, accessory genes (Additional file 1: Figure S3). The core, soft-core and accessory genomes refer to the set of genes for all 60 strains, 57–59 strains and the remaining 2–56 strains, respectively. There was no soft-core genome identified in the data set. In the flagellar assembly pathway, the genes encoding flagellar motor rotation proteins (*MotA* and *MotB*), flagellin filament structural proteins (*FliC*), flagellar cap proteins (*FliD*), flagellar hook-associated proteins (*FlgL* and *FlgK*), and flagellar secretion chaperone proteins (*FliS*), were found in the core genome of *C. difficile* strains. On the other hand, genes encoding flagellar hook protein (*FlgE*), flagellar basal-body rod modification protein (*FlgD*), flagellar basal-body rod protein (*FlgB*, *FlaC*, and *FlaG*), flagellar hook-basal body complex protein (*FliE*), flagellar M-ring protein (*FliF*), flagellar motor switch protein (*FliG*, *FliM* and *FliN*), flagella biosynthesis protein (*FlhA* and *FlhB*), and flagella assembly protein (*FliH*) and flagellar biosynthetic protein (*FliQ*), were found in the accessory genome of *C. difficile* strains. These results indicate that these genes are well conserved among strains and that flagellar construction as well as attachment and invasion of intestinal epithelial cells, are essential for *C. difficile* infection [72, 73]. Given that flagella motility can affect adhesion and colonization of intestinal epithelial cells, *C. difficile* flagella contribute to pathogenicity and result in mucosal damage and inflammatory responses in the host [74].

### PaLoc and CDT locus (CdtLoc) of *C. difficile*

**Fig. 4:**
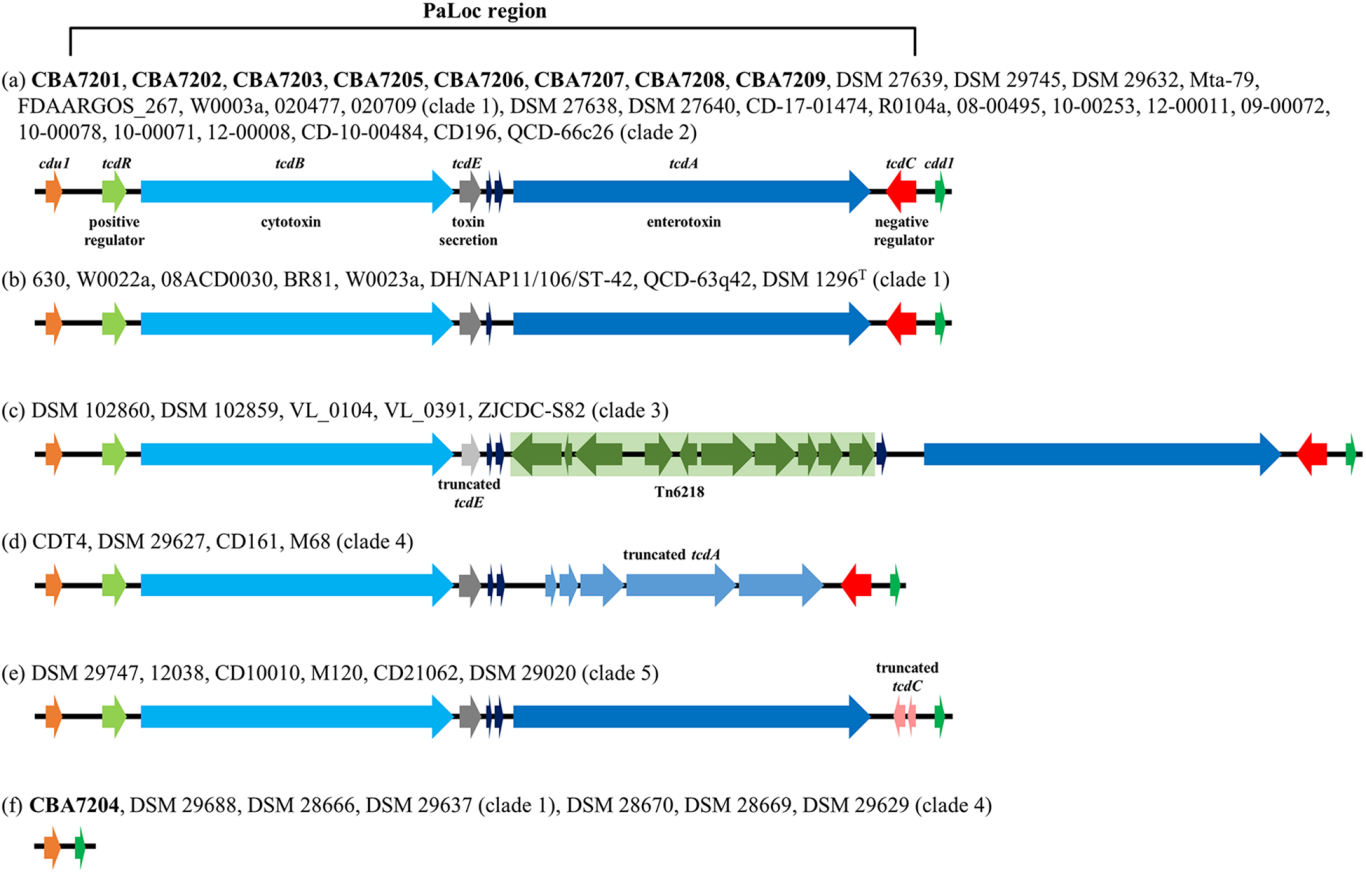
Schematic representation of the PaLoc region and lateral genes. The PaLoc consisting of the *tcdR*, *tcdB*, *tcdE*, *tcdA*, and *tcdC* genes in this order. **a**–**f** PaLoc with two hypothetical protein-coding genes between the *tcdE* and *tcdA* genes (**a**); one hypothetical protein-coding gene between *tcdE* and *tcdA* genes (**b**); one or two hypothetical protein-coding genes and the mobile genetic elements (MGEs) Tn6218 between *tcdE* and *tcdA* gene (**c**); five truncated *tcdA* genes (**d**); two truncated *tcdC* genes (**e**); and non-toxigenic strains (**f**). Strains CBA7201–CBA7209 isolated from Korean patients with CDI are highlighted in bold

Toxins A and B are encoded by the *tcdA* (enterotoxin) and *tcdB* (cytotoxin) genes located on a chromosomal region called the PaLoc (19.6 kb) [75]. The PaLoc structure consists of the *tcdA* and *tcdB* genes sandwiched between the *tcdR* (positive regulator) and *tcdC* (negative regulator) genes, with the *tcdE* gene (toxin secretion) located between the two toxin genes (Fig. 4). Among the 60 strains, all possessed these toxin genes except for the seven non-toxigenic strains (CBA7204, DSM 29688, DSM 28666, DSM 29637, DSM 28670, DSM 28669, and DSM 29629). However, the sequence similarity and structural differences were strain-dependent. All Korean *C. difficile* strains except for strain CBA7204 were found to possess the structure including all of the genes of PaLoc region with two hypothetical protein-coding genes between *tcdE* and *tcdA* genes, as shown in Fig. 4a; 30 strains including 16 strains belonging to MLST clade 1 and 14 strains belonging to clade 2, had the same PaLoc region structure. The remaining strains of clade 1 including strain 630, had only one hypothetical protein-coding gene (Fig. 4b). The insertion of the mobile genetic elements (MGEs) Tn6218, which contains an macrolide, lincosamide and streptogramin-associated antibiotic resistance gene [51, 66] between the truncated *tcdE* and *tcdA* genes, is a common characteristic of clade 3 *C. difficile* strains (Fig. 4c) [76]. Some clade 4 strains face issues with enterotoxin expression due to truncated *tcdA* genes (Fig. 4d) [1]. Meanwhile, clade 5 *C. difficile* strains have a truncated *tcdC* gene, indicating difficulties in suppressing toxin production; thus these strains may become hypervirulent *C. difficile* strains (Fig. 4e) [24]. Four strains including strain CBA7204 belonging to clade 1 and three strains belonging to clade 4 were identified not to have the PaLoc genes (Fig. 4f).

**Fig. 5:**
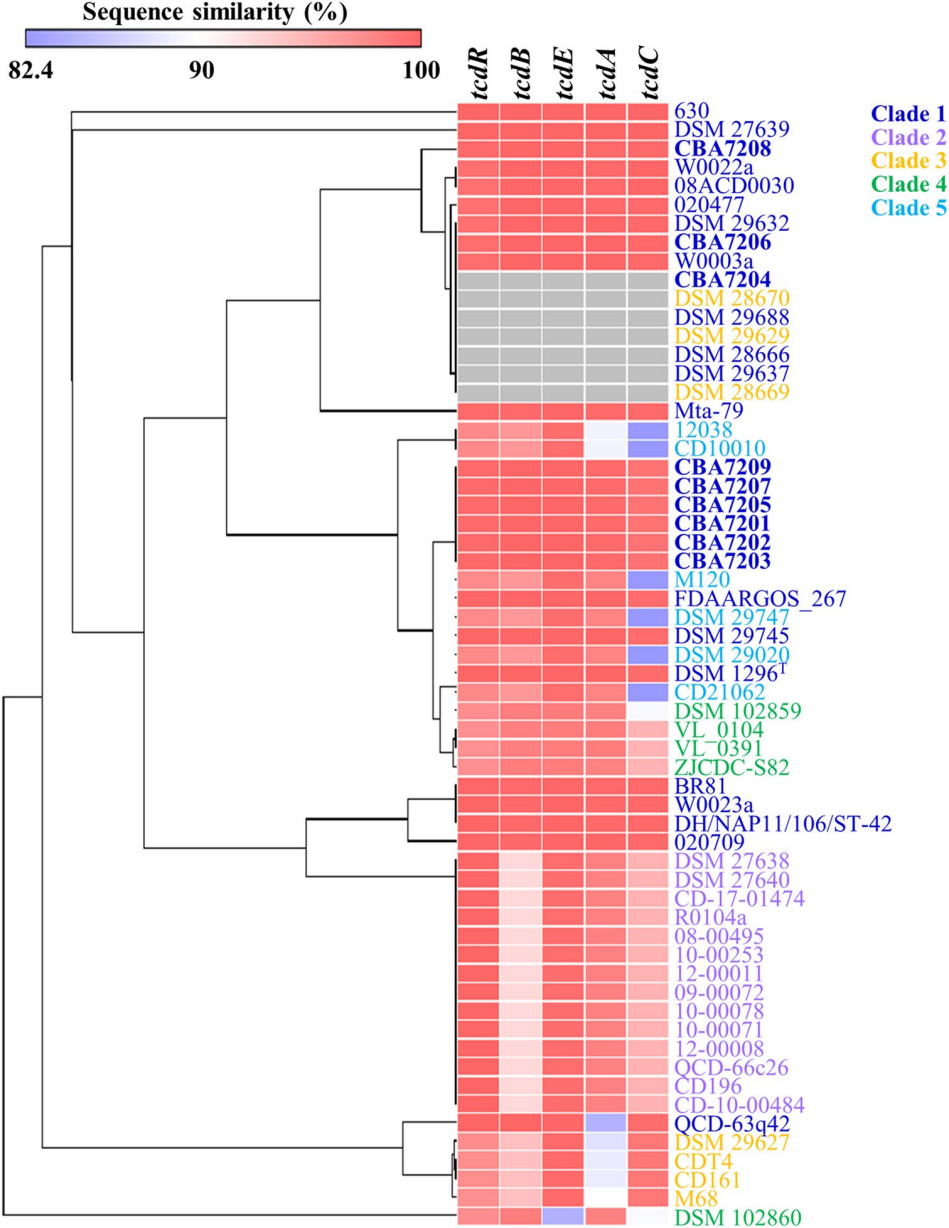
Heat map and hierarchical cluster analysis of PaLoc region genes. A heat map illustrating the sequence similarity. Strains CBA7201–CBA7209 isolated from Korean patients with CDI are highlighted in bold. Strain names of *C. difficile* strains are indicated differently depending on the MLST clade: navy blue (clade 1), purple (clade 2), yellow (clade 3), green (clade 4), and sky blue (clade 5). Strains CBA7201–CBA7209 isolated from Korean patients with CDI are highlighted in bold

Sequence similarities of PaLoc genes between strain 630 that is the most used reference strain in *C. difficile* genomic analysis and other strains, were compared [21]. Compared with *C. difficile* 630, strains CBA7201, CBA7202, CBA7203, CBA7205, CBA7207, and CBA7209 exhibited similar PaLoc sequence similarities and formed a unique group (Fig. 5). Among the strains belonging to clade 1, except for the non-toxigenic strains and strain QCD-63q42, each PaLoc gene showed a similarity of 99.3% or more. Strain QCD-63q42 had 84.7% similarity of *tcdA* gene with strain 630. As shown in Fig. 4, the strains in clade 2 had the same gene size as the strains in clade 1, but the similarity values of *tcdB* (93.5%), *tcdA* (98.5%), and *tcdC* (95.7%) genes were slightly lower than those in clade 1. In the case of the strains belonging to clade 3, the sequence similarities of *tcdR* (97.7–97.8%), *tcdB* (98.6–98.7%), *tcdA* (98.5–98.8%), and *tcdC* (90.7–95.7%) genes were found. In the case of *tcdE* gene of the strains in clade 3, the gene size was 424 bp (Fig. 4c), which was shorter than the gene (501 bp) of strain 630, but the similarity of the gene was 98.8%, which was relatively high compared to the rest of the PaLoc genes. In the case of clade 4, 3 of the 7 strains were non-toxigenic (Fig. 4f) and the remaining strains showed low similarity value of *tcdR* (97.8%) and *tcdB* (94.9%) genes. The truncated *tcdA* gene of clade 4 had a similarity value of 88.6–89.6% (Fig. 4d). In the case of clade 5, it was confirmed that the truncated *tcdC* gene had a particularly low similarity with a value of 82.4% (Fig. 4e).

**Fig. 6:**
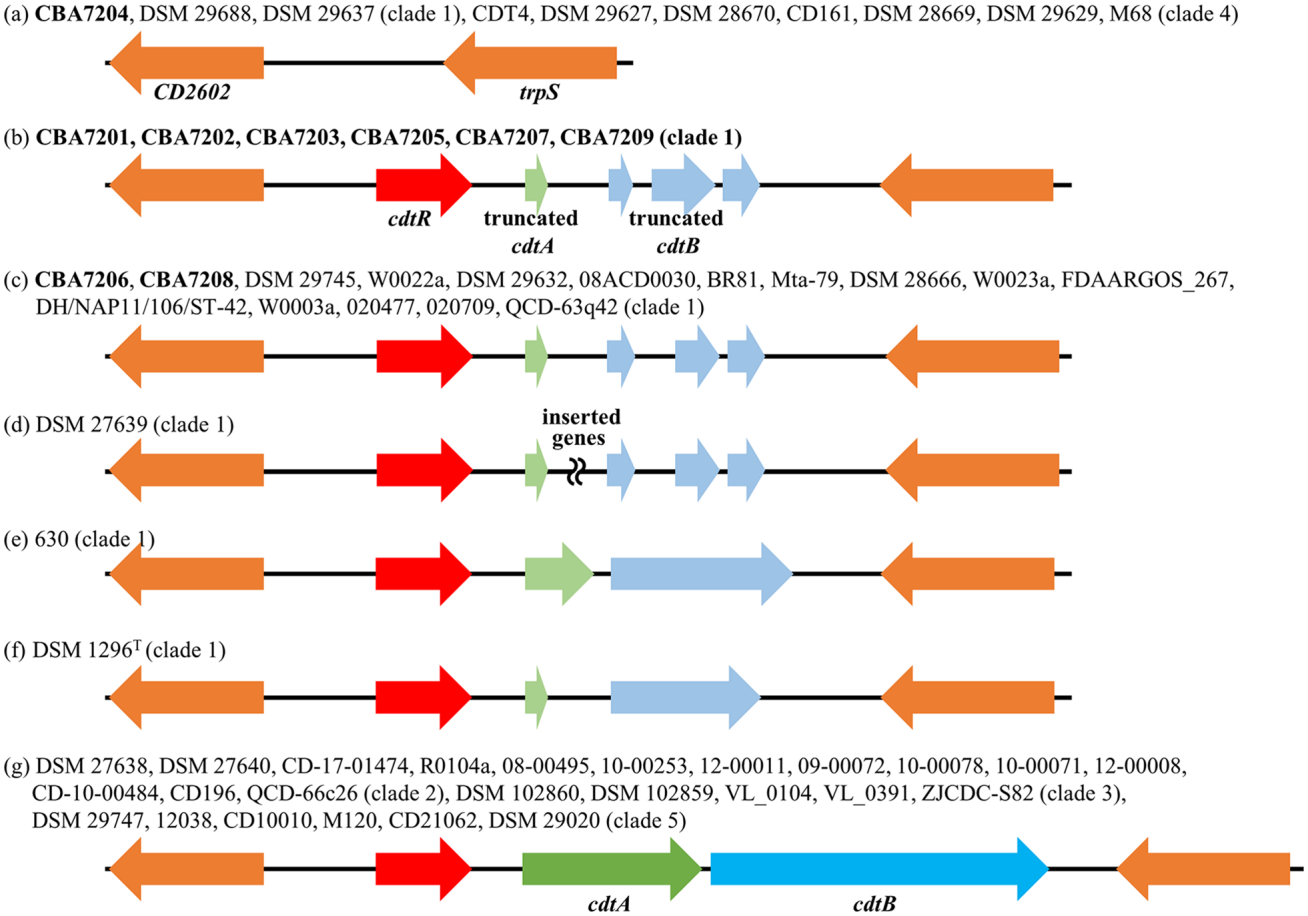
Schematic representation of the CDT region and lateral genes. Gene organization of the region without the *cdt* gene **a** and of binary toxin-negative strains **b**–**f** and binary toxin gene-positive strains (**g**). Strains CBA7201–CBA7209 isolated from Korean patients with CDI are highlighted in bold

The actin-ADP-ribosylating toxin is located on a chromosomal region CdtLoc. *C. difficile* strains carrying the CDT gene may exhibit stronger virulence due to interaction with the existing toxins A and B [16–19]. The structure of the CdtLoc region consists of a binary toxin regulatory gene (*cdtR*) and binary toxin genes (*cdtA* and *cdtB*). These three genes differ in sequence similarity and structure depending on the *C. difficile* strains (Fig. 6). Some clade 1 strains and all clade 4 strains were not found to possess a CdtLoc region (Fig. 6a), while other clade 1 strains may show difficulty in producing the toxin due to gene truncation (Fig. 6b–f); for instance, strain DSM 27639 has an inserted gene (approximately 30 kb) between the *cdtA* and *cdtB* genes (Fig. 6d) [77]. However, all strains belonging to clade 2, 3, and 5 possessed an intact CdtLoc region (6.2 kb), indicating the normal expression of toxin-producing genes (Fig. 6g).

### Common features of the six *C. difficile* strains isolated from Korean patients with CDI

Finally, we identified several common features among *C. difficile* CBA7201, CBA7202, CBA7203, CBA7205, CBA7207, and CBA7209 isolated from Korean patients with CDI. These strains were not single clones, as evidence by differences between the strains confirmed via phylogenetic analysis; core, accessory, and unique gene analysis; and OrthoANI values (Fig. 3 and Additional file 2: Table S1 and S2). Nevertheless, they share several common features. All the six strains belong to clade 1 and ST17 (Table 2) and possess similar antibiotic-resistance genes (Table 3). In addition, they exhibited similar toxin gene expression in terms of PaLoc and CdtLoc structure (Figs. 4, 5, 6). Interestingly, the cephalosporins such as Zenocef, Cetrazol, Pacetin, and Cefazolin, were commonly prescribed to the six patients with CDI, from whom these six strains were isolated (Table 1). However, the cephalosporin resistance gene was not detected in all six strains isolated from cephalosporin-containing medium (Table 3). In a previous case study, the cephalosporins presented a risk factor to patients with CDI, and the decrease in cephalosporin prescription rate was related to a decrease in diarrhea cases associated with *C. difficile* [9, 78–81]. Therefore, further studies are needed to elucidate the association among antibiotics, *C. difficile* strains, and patients with CDI.

## Conclusion

In this study, we investigated the genomic, phylogenetic, functional, and pathogenic features of nine *C. difficile* strains isolated from Korean patients and performed a comparative genomic analysis with other strains isolated from various countries. Along with the identified genomic features of Korean *C. difficile* isolates, accumulation of more whole-genome sequence information of diverse *C. difficile* strains could serve as basic information for CDI prevention and treatment in Korea.

## Supplementary Information

**Additional file 1:Fig. S1.** Pan- and core-genome box plot of 60 *C. difficile* strains with standard deviations. The pan-genome represents the total set of genes of the 60 *C. difficile* strains, while the core-genome represents the common genes across all genomes. **Fig. S2.** *In silico* DNA-DNA hybridization (DDH) analyses showing the pair-wise relatedness of 60 *C. difficile* strains and two reference strains (*C. mangenotii* DSM 1289^T^ and *Clostridium hiranonis* TO-931). Strains CBA7201–CBA7209 isolated from Korean patients with CDI are highlighted in bold. The hierarchical clusters represented by dendrograms were constructed by simple linkage of the *in silico* DDH values. The vertical bar on the right side of the figure indicates the MLST clade to which each *C. difficile* strain belongs. **Fig. S3. **Diagram of the structural components involved in *C. difficile* flagella assembly defined by the number of KEGG orthology genes identified from the genomes of 60 *C. difficile* strains. Flagella assembly genes belonging to the core genome of the 60 *C. difficile* strains are indicated in red; flagella assembly genes belonging to the accessory genome identified from 2–56 genomes are indicated in blue.

**Additional file 2: Table S1.** The number of the core-genes, accessory-genes (present in more than two strains), and unique-genes present in 60 *C. difficile* strains. **Table S2. **OrthoANI (average nucleotide identity) analyses showing the pair-wise relatedness of 51 *C. difficile* strains and two reference strains (*C. mangenotii* DSM 1289T and *Clostridium hiranonis* TO-931) for nine *C. difficile* strains isolated from Korea. **Table S3.** Lists of antibiotics resistance gene present in 60 *C. difficile* strains.

## Data Availability

The complete genome data of strain CBA7201–CBA7209 has been deposited in DDBJ/EMBL/GenBank, with accession numbers QKRF00000000, QLNX00000000, QKRE00000000, CP029566, QLNY00000000, QLNZ00000000, QLOA00000000, QKRD00000000, and QLOB00000000, respectively.
