# Supplementary material for: Genomic characterization of nine Clostridioides difficile strains isolated from Korean patients with Clostridioides difficile infection
Source: Gut Pathog. 2021 Sep 16;13:55. doi: 10.1186/s13099-021-00451-3 (PMC8447795; doi:10.1186/s13099-021-00451-3)

**Additional file 1: Fig. S1.** Pan- and core-genome box plot of 60 *C. difficile* strains with standard deviations. The pan-genome represents the total set of genes of the 60 *C. difficile* strains, while the core-genome represents the common genes across all genomes.


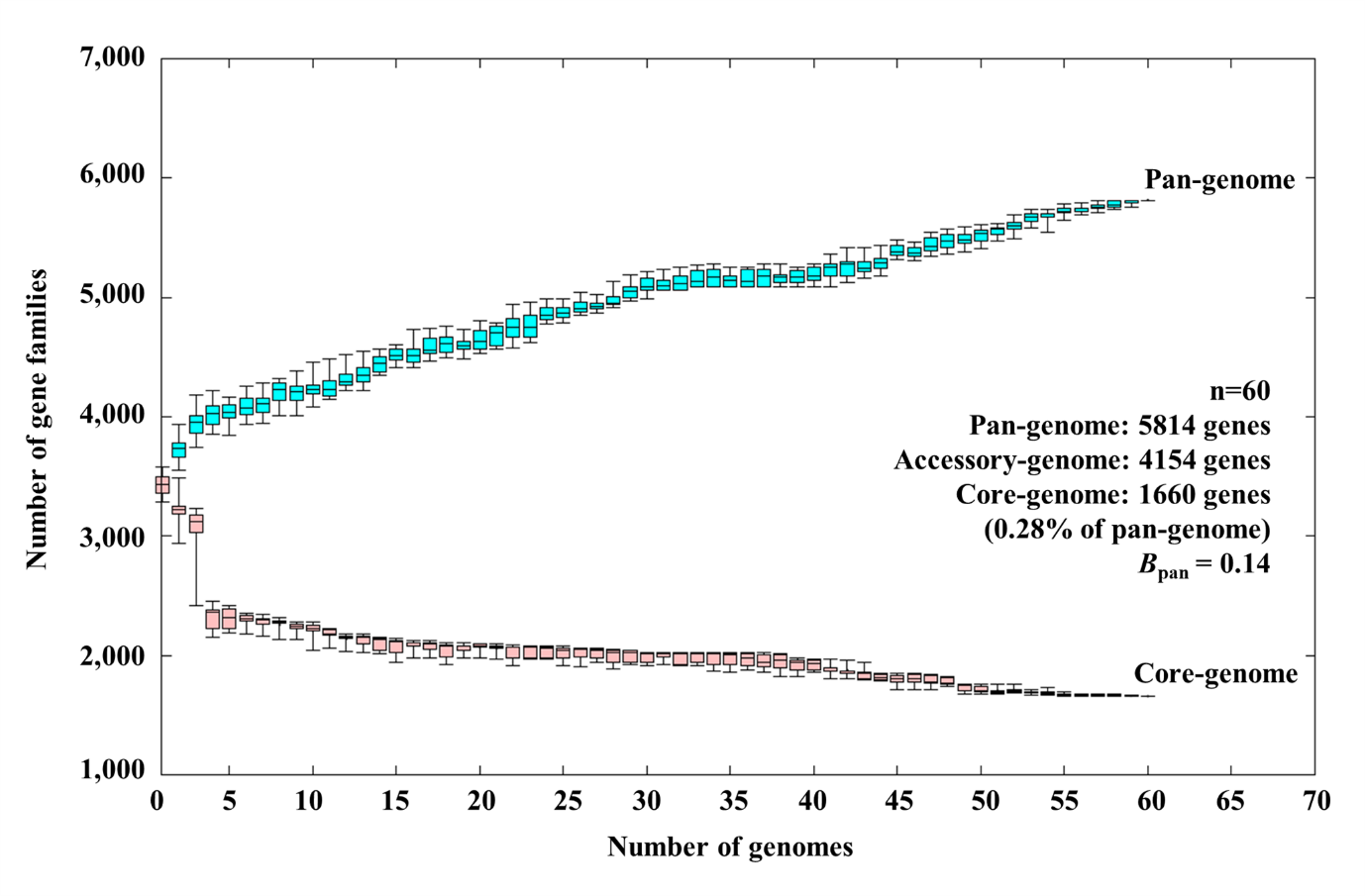


**Additional file 1: Fig. S2.** *In silico* DNA-DNA hybridization (DDH) analyses showing the pair-wise relatedness of 60 *C. difficile* strains and two reference strains (*C. mangenotii* DSM 1289^T^ and *Clostridium hiranonis* TO-931). Strains CBA7201–CBA7209 isolated from Korean patients with CDI are highlighted in bold. The hierarchical clusters represented by dendrograms were constructed by simple linkage of the *in silico* DDH values. The vertical bar on the right side of the figure indicates the MLST clade to which each *C. difficile* strain belongs.


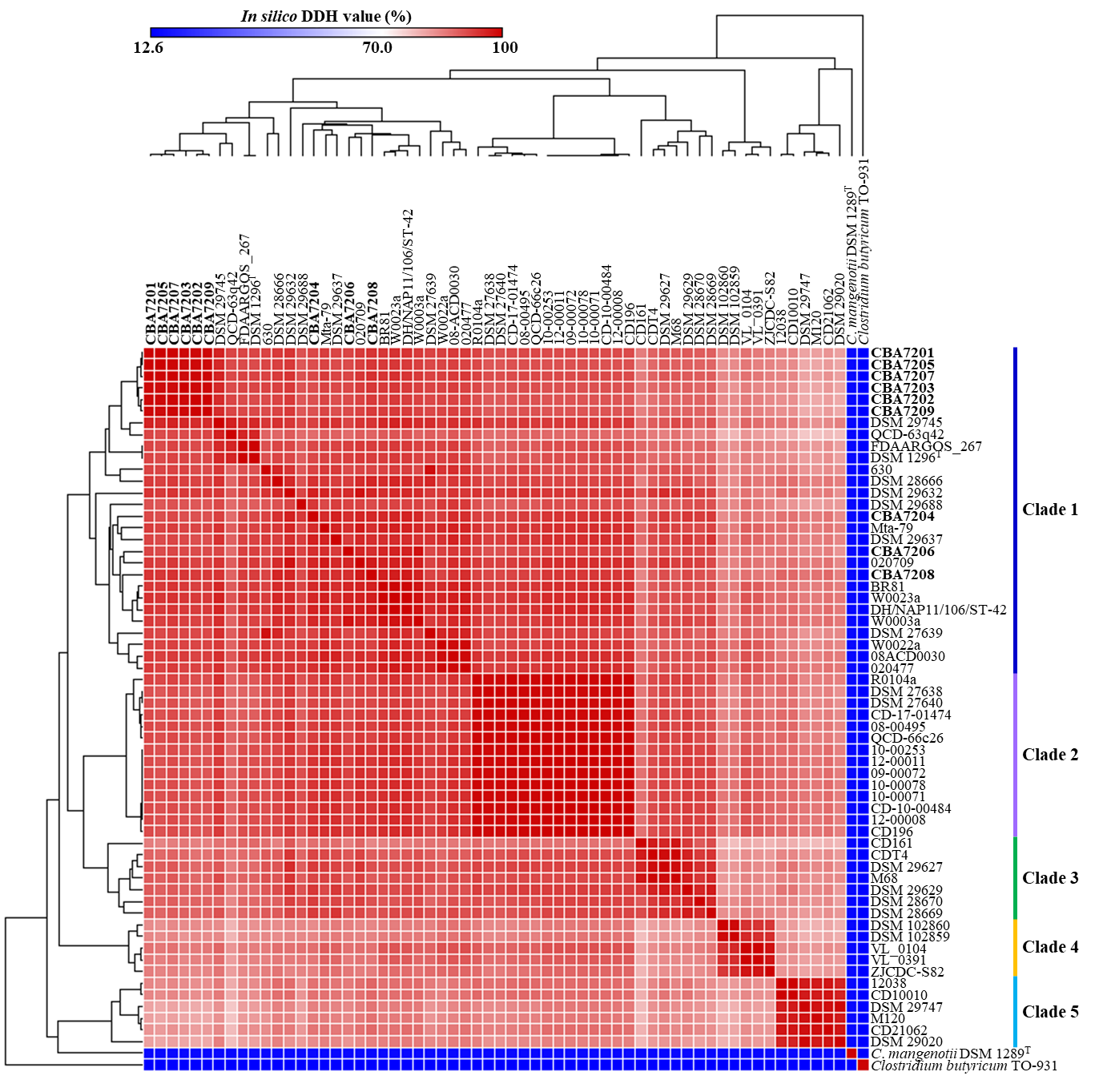


**Additional file 1: Fig. S3.** Diagram of the structural components involved in *C. difficile* flagella assembly defined by the number of KEGG orthology genes identified from the genomes of 60 *C. difficile* strains. Flagella assembly genes belonging to the core genome of the 60 *C. difficile* strains are indicated in red; flagella assembly genes belonging to the accessory genome identified from 2–56 genomes are indicated in blue.


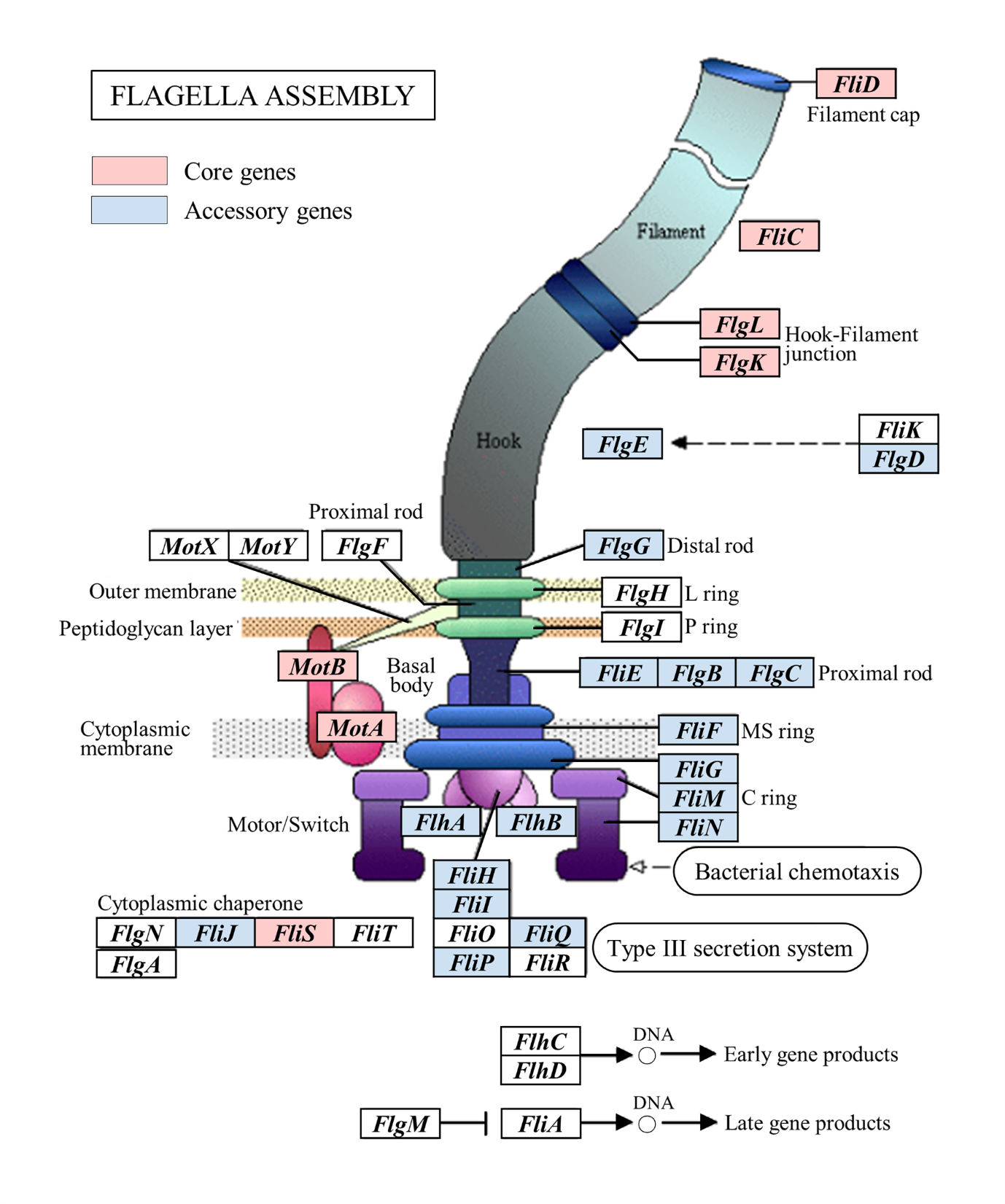

Supplement: Supplementary file 1 — Additional file 1:Fig. S1. Pan- and core-genome box plot of 60 C. difficile strains with standard deviations. The pan-genome represents the total set of genes of the 60 C. difficile strains, while the core-genome represents the common genes across all genomes. Fig. S2. In silico DNA-DNA hybridization (DDH) analyses showing the pair-wise relatedness of 60 C. difficile strains and two reference strains (C. mangenotii DSM 1289T and Clostridium hiranonis TO-931). Strains CBA7201–CBA7209 isolated from Korean patients with CDI are highlighted in bold. The hierarchical clusters represented by dendrograms were constructed by simple linkage of the in silico DDH values. The vertical bar on the right side of the figure indicates the MLST clade to which each C. difficile strain belongs. Fig. S3. Diagram of the structural components involved in C. difficile flagella assembly defined by the number of KEGG orthology genes identified from the genomes of 60 C. difficile strains. Flagella assembly genes belonging to the core genome of the 60 C. difficile strains are indicated in red; flagella assembly genes belonging to the accessory genome identified from 2–56 genomes are indicated in blue. [file 13099_2021_451_MOESM1_ESM.docx]
